# NiMoS-Modified Carbon Felt Electrode for Improved Efficiency and Stability in a Neutral S/Fe Redox Flow Battery

**DOI:** 10.3390/molecules30061219

**Published:** 2025-03-08

**Authors:** Dan Mei, Bowen Liu, Haiqing Ma, Zhaoguo Zhang, Fan Wu, Yanan Chen, Jawad Ali, Futang Xing, Liangbin Xiong

**Affiliations:** 1 College of Resources and Environmental Engineering, Wuhan University of Science and Technology, Wuhan 430081, China; meidan@wust.edu.cn (D.M.); liubowen@wust.edu.cn (B.L.);; 2 School of Environment and Biological Engineering, Wuhan Technology and Business University, Wuhan 430065, China; 3 School of Optoelectronic Engineering, Guangdong Polytechnic Normal University, Guangzhou 510665, China

**Keywords:** polysulfide-ferricyanide redox flow battery, NiMoS catalyst, carbon felt electrode, electrochemical kinetics, cycling stability

## Abstract

Polysulfide-ferricyanide redox flow batteries (PFRFBs) are gaining significant attention in long-duration energy storage for their abundant availability and environmental benignity. However, the sluggish kinetics of the polysulfide redox reactions have tremendously constrained their performances. To address this issue, we developed a NiMoS catalyst-modified carbon felt (NiMoS-CF) electrode, which significantly accelerates the electrochemical reaction rates and enhances the cycling stability of PFRFB. Our PFRFB system, integrated with the NiMoS-CF electrode, exhibited an energy efficiency of 70% and a voltage efficiency of 87%, with a remarkable doubling of its cycle life as opposed to the pristine carbon felt (CF) electrode at a current density of 40 mA cm^−2^. Notably, during 2500 cycles of charge–discharge testing, we achieved an average coulombic efficiency exceeding 99%. These improvements in PFRFB performance can be attributed to the NiMoS-CF electrode’s large surface area, low resistance, and robust redox activity. This study offerings a novel approach for enhancing the electrochemical reaction kinetics and cycling stability in PFRFBs, laying a scientific foundation in the applications of practical PFRFBs for next-generation energy storage.

## 1. Introduction

As environmental pollution escalates and fossil fuel reserves diminish, there is a growing focus on harnessing eco-friendly and sustainable energy alternatives, such as solar and wind energy. To achieve the successful adoption of these intermittent resources, developing the long-duration energy storage systems is critically important [1]. Redox flow batteries (RFBs) have become an ideal solution for long-term energy storage due to their versatile design, high efficiency, durable cycle life, and excellent deep discharge abilities [2,3,4]. The current widely used flow battery is all-vanadium redox flow batteries (VRFBs), it still faces low energy density and cost fluctuations due to low vanadium solubility and limited resources [5,6,7,8]. Optimizing electrochemical performance, increasing energy and power density, lowering internal resistance, and improving electrolyte cycle stability are all critical for the development of RFBs for long-term energy storage.

Current research and development focuses on low-cost, high-energy density, and long-term RFBs [9]. Emerging neutral polysulfide-ferricyanide redox flow batteries (PFRFBs) are gaining popularity because of their high energy density (260 Wh L^−1^) and inexpensive cost (<19.26 $ kW h^−1^). Iron is abundant and affordable for large-scale energy storage [10,11,12]; Sulfur’s multielectron redox reaction and high theoretical energy density, when paired with iron, boost active ingredient usage and electrochemical performance [13,14], especially in S/Fe RFBs [15,16]. However, the battery performances of S/Fe RFBs including the voltage efficiency, energy efficiency, power density, and cycle life, are constrained by the sluggish kinetics of the polysulfide redox reactions. In the charging process, the large overpotential (>500 mV) and low energy efficiency (<50%) are resulted from the electrochemical reduction of polysulfide even with the moderate current densities (10–20 mA cm^−2^). This is a common challenge in polysulfide-based battery systems, which can be found in other sodium and lithium-sulfur batteries.

To facilitate the reduction of polysulfide, adding chemicals [17,18,19,20], electrolytes [21,22,23], etc. can increase its electrochemical characteristics and stability. Notably, redox processes that involve electrochemically active couples predominantly occur at the electrodes of RFBs. The dynamics of these redox reactions, which directly affect the performance of the battery, are affected by various factors associated with the physical and chemical characteristics of the electrodes, as well as their electrochemical behavior. These factors include surface roughness, wettability, durability, surface area, electrical conductivity, and the electrochemical activity. Several approaches have been investigated to enhance the kinetics of polysulfide ions. For example, Lan [13] improved the mass transfer and redox behavior of aqueous polysulfides by employing a nanostructured, self-assembled, hierarchically porous Co and N dual-doped carbon (OHP-Co/NC) as an electrocatalyst in an electrocatalytic reactor, resulting in a high power density of 110 mW cm^−2^ and 99.7% capacity retention, but the interfacial force between the carbon carrier and the active center in the catalyst is weak, which may result in the loss or inactivation of its active components, compromising the catalyst’s long-term stability. Sulfides have higher electrical conductivity than oxides, which allows for more efficient charge transfer [24], hance various metal sulfides like mesocrystalline NiS_2_ [25], CoS_2_/CoS [26,27], CuS [28] have been employed to boost the kinetics of polysulfide ions. Motivated by these pioneered studies, this work aims to develop novel S/Fe RFB system with accelerated kinetics of polysulfide redox reactions and long cycling life. It has not been reported, though, that NiMoS—a catalyst with strong stability and activity in electrocatalytic water splitting and catalytic hydrogenation and oxidation reactions [26,29,30]—is used in polysulfide-ferricyanide redox flow batteries. Here, we introduce NiMoS catalysts modified carbon felt (NiMoS-CF) as an anode electrode, capitalizing on the catalytic properties of metal sulfides to enhance polysulfide redox processes. NiMoS-CF demonstrated good charge transfer and catalytic performance for polysulfide redox processes. By directly growing electrocatalytic materials on carbon felt (CF), we created modified electrodes without binders, avoiding the negative impacts of adhesives. The PFRFB, equipped with the NiMoS-CF electrode, exhibited improved electrochemical reaction rates, energy density and cycling stability, with a 70% energy efficiency, a coulombic efficiency of over 99% and the cycle life of 2500 cycles at 40 mA cm^−2^. In contrast, the PFRFB with pristine CF as the negative electrode ended after 1300 cycles, leading to a faster capacity decline.

## 2. Results and Discussion

**Figure 1:**
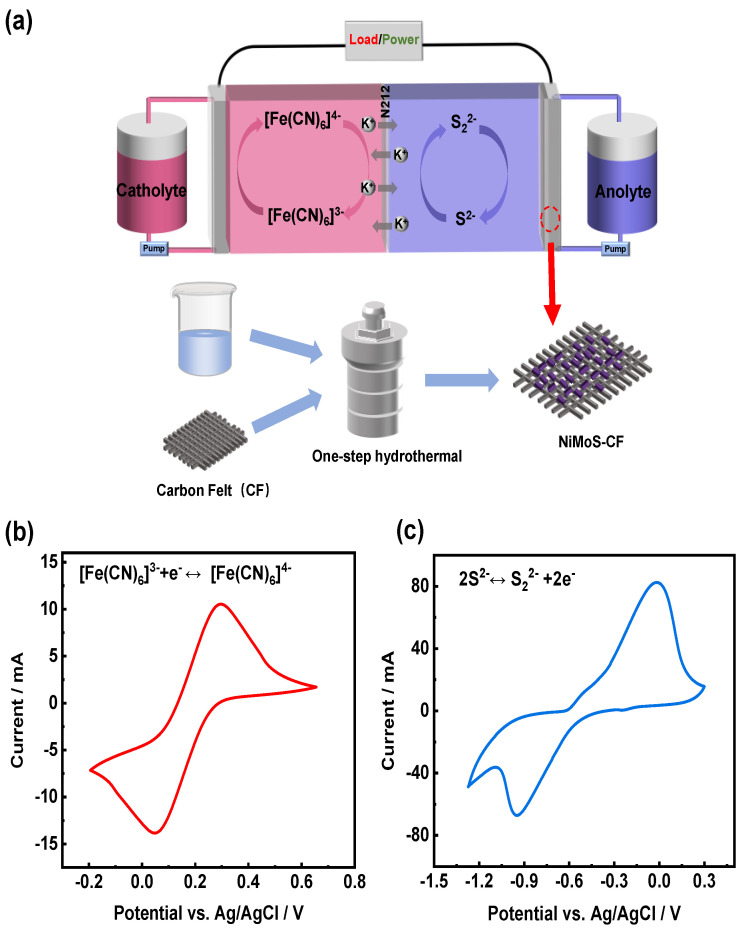
(**a**) Schematic illustration of the S/Fe redox flow battery system; CV curves of (**b**) ferricyanide/ferrocyanide and (**c**) sulfide/polysulfide in 1.0 M KCl electrolyte at 10 mV s^−1^.

Figure 1a illustrates the configuration of S/Fe redox flow battery, in which K_2_S and K_3_[Fe(CN)]_6_ are utilized as the anolyte and catholyte, respectively. The catholyte and anolyte are composed of the redox couples [Fe(CN)_6_]^4−/3−^ and S_2_^2−^/S^2−^, respectively, with KCl as the supporting electrolyte. As demonstrated in the preparation process of the NiMoS-CF electrode, a one-step hydrothermal process was employed to synthesize the NiMoS catalyst on the CF, avoiding the negative impacts of the adhesive nafion which is commonly used. It is hypothesized that the catalyst can significantly extend the surface area of the original CF, provide new reaction sites, and improve the conductivity of the electrode [31]. The electrochemical reversibility of the species involved in the redox reactions. was assessed using CV tests. The following redox reactions are represented by one pair of redox peaks in the respective positive and negative electrolyte. As shown in Figure 1b,c, the reversibility of the reaction is usually judged by the symmetry of the curve [32], the redox couples [Fe(CN)_6_]^4−/3−^ and S_2_^2−^/S^2−^ show good reversibility and exhibit well-defined redox peak.

$${{\left[Fe(CN\right)}_{6}]}^{3-}+e^{-}↔{{\left[Fe(CN\right)}_{6}]}^{4-}\;\;\;E^{0}=0.46\;V\;vs.SHE$$

$${2S}^{2-}↔{S_{2}}^{2-}+2e^{-}\;\;\;E^{0}=-0.36\;V\;vs.SHE$$

### 2.1. Structure and Morphology of NiMoS-CF Electrode

**Figure 2:**
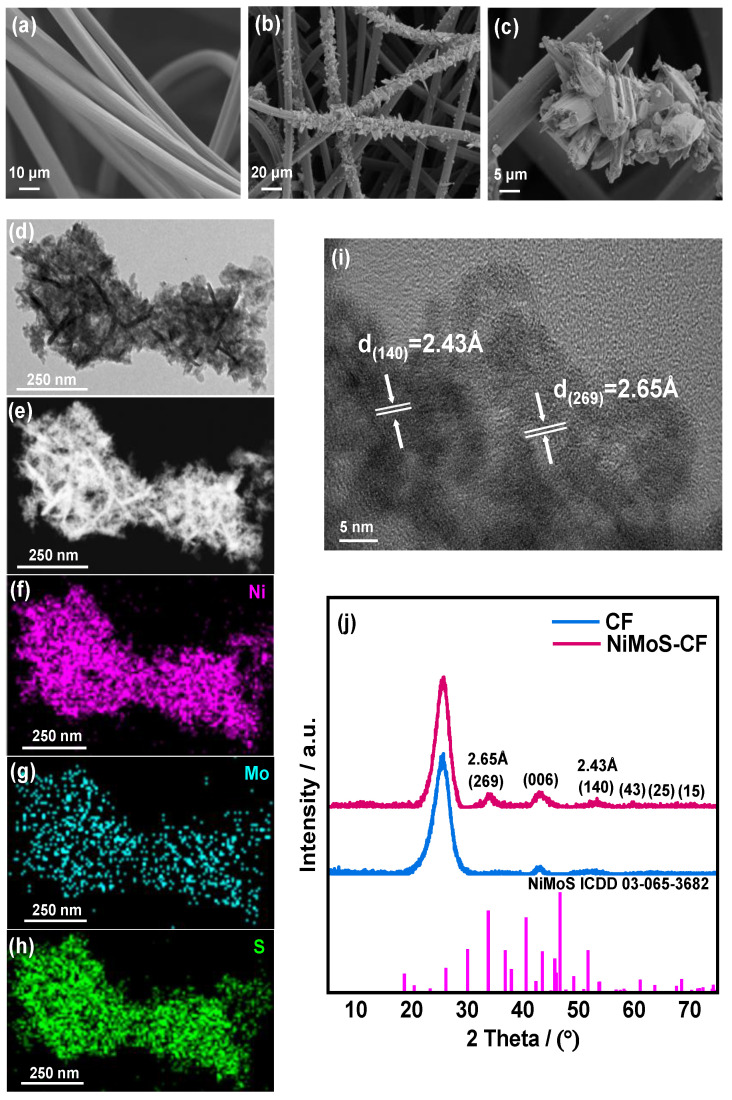
SEM images of the (**a**) pristine CF and (**b**,**c**) NiMoS-CF; (**d**) TEM image of NiMoS-CF; (**e**) HAADF-STEM image of NiMoS-CF; (**f**–**h**) EDS mapping of Ni, Mo and S; (**i**) HR-TEM image of NiMoS-CF; (**j**) XRD patterns of pristine CF and NiMoS-CF.

The SEM photographs reveal significant morphological distinctions between pristine CF and NiMoS-CF, indicating effective nucleation and growth of NiMoS on the surface. Specifically, the pristine CF (Figure 2a) exhibits a clean and smooth surface, whereas the NiMoS-CF (Figure 2b,c), after 12 h of hydrothermal treatment, displays a rough surface. Consequently, the electrode surface becomes relatively rough and this morphology not only provides additional active sites for redox reactions but promotes close contact with the electrolyte, thereby accelerating the kinetics of these reactions [33,34]. In the TEM image shown in Figure 2d, the distinct nanorods were observed. The spatially uniform distribution of Ni, Mo, and S elements can be revealed by HAADF-STEM analysis coupled with EDS mapping (Figure 2e–h). In Figure 2i, the HR-TEM image exhibits obvious lattice fringes with interplanar distances of 2.65 Å and 2.43 Å, aligning with the d-spacing of (269) and (140) planes of NiMoS. These crystalline planes are evident from the XRD patterns. Compared with the pristine CF and the standard NiMoS patterns, not only the diffraction peak from CF, but also the main peak of NiMoS can be obviously observed in the prepared NiMoS-CF, which confirms that the NiMoS catalyst is successfully grown on the CF (Figure 2j).

**Figure 3:**
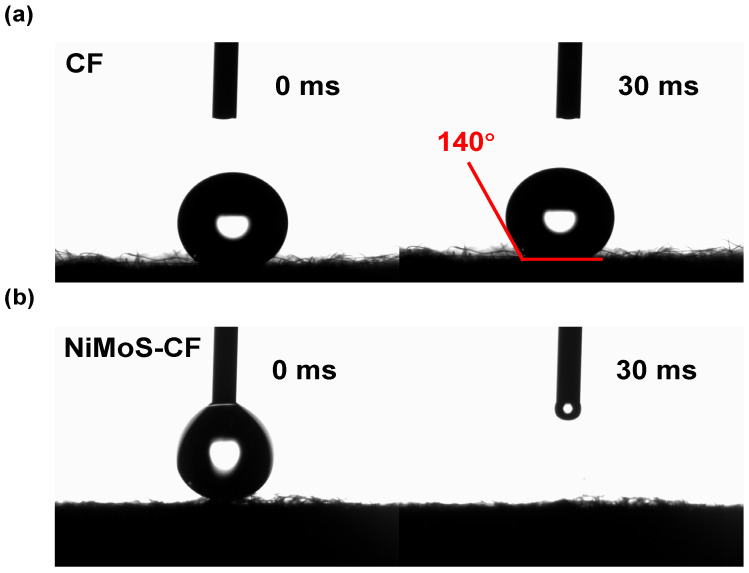
Contact angle measurements of (**a**) pristine CF and (**b**) NiMoS-CF.

Also, significant differences in hydrophilicity were identified before and after the NiMoS modification on the CF. The contact angle of the pristine CF was 140° (Figure 3a), indicating that the surface of the material was lyophobic, while NiMoS-CF rapidly absorbed droplets during the test, showing hydrophilicity (Figure 3b). Specifically, the increased wettability facilitates better electrolyte accessibility and more efficient mass transport of reactants to the active sites, reduced the charge transfer resistance.

The XRD patterns and XPS survey spectrum of NiMoS catalyst can be found in Figures S2 and S3. The unindexed peaks in the XRD pattern are attributed to the presence of minor secondary phases and impurities in the catalyst such as MoS_2_ [35]. These phases likely arise from incomplete crystallization or residual precursors during the synthesis process. XPS analysis confirmed the presence of Ni, Mo, and S elements within the catalyst. The Ni 2p XPS spectra display two shakeup satellites (labeled Sat.) at 859.9 and 877.6 eV, along with two spin-orbit doublets, corresponding to Ni 2p_3/2_ (Ni^2+^ at 853.5 and Ni^3+^ at 855.2 eV) and Ni 2p_1/2_ (Ni^2+^ at 871.3 and Ni^3+^ at 873.3 eV) [36,37,38]. The Mo 3d XPS spectra are shown in Figure S3c, with peaks at 232.2 eV and 235.2 eV corresponding to Mo 3d_5/2_ and Mo 3d_3/2_, respectively [39]. The spectra of S 2p is illustrated in Figure S3d. Figure S3d illustrates the S 2p XPS spectra, where the peaks at 163.5 eV and 162.3 eV are assigned to 2p_1/2_ and 2p_3/2_ of S^2−^, and the peak at 168.6 eV is indicative of sulfate species [40,41].

### 2.2. Catalytic Performance of NiMoS-CF Electrode for Sulfur Ions

#### 2.2.1. Electrochemistry Tests

**Figure 4:**
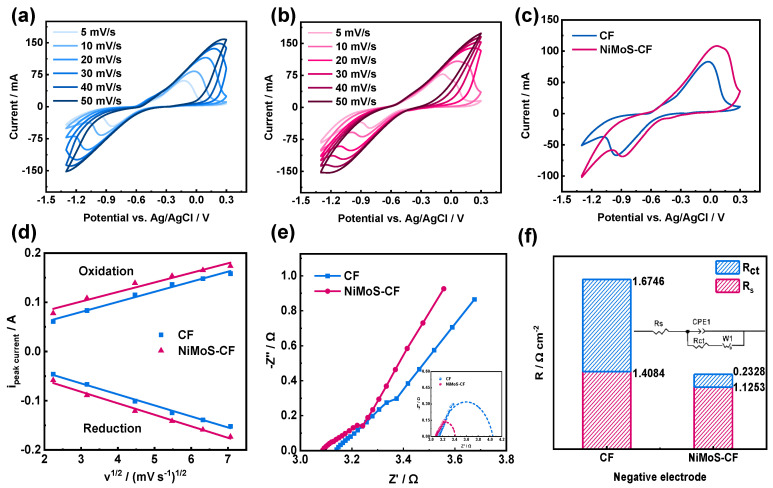
CV profiles for (**a**) pristine CF and (**b**) NiMoS−CF at various scan rates; (**c**) CV curves of pristine CF and NiMoS−CF at a scan rate of 10 mV s^−1^; (**d**) peak currents fitting lines for the S_2_^2−^/S^2−^ redox pair on pristine CF and NiMoS−CF plotted against the square root of the scan rates; (**e**) Nyquist plots from the resulting EIS of pristine CF and NiMoS−CF with fitting curves; (**f**) comparison of resistance values derived from the Nyquist plots.

The electrochemical response of CF and NiMoS-CF to the redox reaction of K_2_S was investigated in Figure 4a,b, detailed CV data are shown in Table S1, respectively. At the equilibrium potential of −0.455 V, the major redox reaction is attributed to the S_2_^2−^/S^2−^ pairs [42]. At all test rates, the current densities of the main redox peaks on NiMoS-CF are higher than those on pristine CF. With a sweep rate of 10 mV s^−1^, both pristine CF and NiMoS-CF present distinct redox peaks (Figure 4c). These findings suggest that the surface coverage of NiMoS enhances the electrochemical activity. The catalyst surface area is a key determinant of electrochemical performance, as indicated in previous studies [28,43,44,45]. The glassy carbon electrode (GCE) provides a flat, well-defined surface area that allows precise evaluation of the intrinsic catalytic activity of NiMoS. To minimize confounding factors such as substrate porosity, mass transport limitations, or uneven catalyst distribution that may arise when directly coating fibrous substrates like CF, replacing a glassy carbon electrode (GCE) for the working electrode (Figure S4), which is less susceptible to external factors and exhibits enhanced conductivity, multiple polysulfide redox peaks were observed (Figure S5). The NiMoS-GCE shows a significant peak-to-peak separation in the CV, this observation can be attributed to the intrinsic limitations of CV in capturing subtle kinetic improvements, especially in systems where substrate effects dominate. The GCE data and carbon felt-based results collectively provide a comprehensive understanding of both catalyst efficiency and scalability. According to the result, a more complex electrochemical reaction occurred among the polysulfides, and the valence states of S ions not only include S_2_^2−^/S^2−^ but may also involve additional polysulfide species, such as S_x_^2−^ (x = 2–8). These redox reactions are directly linked to the energy conversion efficiency of the battery, as demonstrated in various sulfur-based batteries [26,31].

Since the diffusion constant of the active ion is proportional to the ratio of i and $\upsilon^{1/2}$, the linear lines developed for NiMoS-CF exhibit steeper slopes (Figure 4d), suggesting that the transfer process of active ions on CF and NiMoS-CF is controlled by diffusion, and the catalyst changes the diffusion rate rather than the mode of control. The resistance of both pristine CF and NiMoS-CF were characterized through electrochemical impedance spectroscopy (EIS) tests, as illustrated in Figure 4e. Furthermore, the measured charge transfer resistance (R_ct_) of the NiMoS-CF (524 mΩ) was lower than that of the pristine CF (3768 mΩ), showing that the redox kinetics of the S_2_^2−^/S^2−^ pair are enhanced on the NiMoS-CF and significantly accelerates the redox reaction (Figure 4f, Table S2). In addition, in Figure 4e, the fitting curve of the high frequency region is added for better comparison.

**Figure 5:**
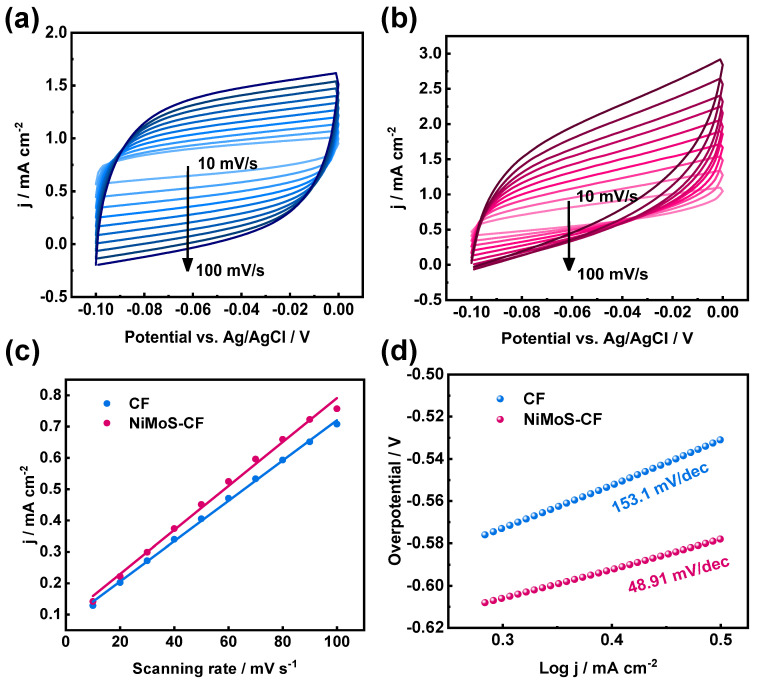
CV profiles for (**a**) pristine CF and (**b**) NiMoS−CF in non-Faraday intervals; (**c**) the corresponding $C_{dl}$ comparison of different electrodes; (**d**) Tafel slopes for different electrodes.

ECSA refers to the Electrochemical Active Surface Area: a higher ECSA value implies that the effective area of the electrode is greater. Furthermore, the Tafel slope was quantitatively evaluated. The lower Tafel slope implies that the kinetics of the electrode are faster and its catalytic activity is higher. In the non-Faraday region, non-faraday capacitance of electrode is positively correlated with ESCA. If the measured capacitance is significantly lower than the theoretical value, it may indicate pore blockage, poor electrical conductivity, or low ion accessibility. In addition, the high double layer capacitance means that charge storage achieves a fast response through the adsorption/desorption of electrolyte ions on the electrode surface. CV curves were obtained by CV testing at different sweep speeds (Figure 5a,b), which were subsequently analyzed to determine the double-layer capacitance ($C_{dl}$) for the different electrodes (Figure 5c). The results of the calculations for ($C_{dl}$), ECSA, and Tafel slopeare summarized in Table 1. These findings demonstrate that NiMoS-CF possesses a larger double-layer capacitance, and the electrochemical area estimated from ($C_{dl}$) is greater than that of pristine CF. The decreased Tafel slope (Figure 5d) also indicates that NiMoS-CF exhibits faster reaction kinetics.

Furthermore, the structural properties of NiMoS catalyst, such as abundant edge active sites, provide excellent catalytic sites that enhance the adsorption and dissociation of sulfur ions, thereby accelerating the rate of redox reactions. During the reaction, sulfur is removed off the catalyst surface at a lower level, and the active metals Mo and Ni become unsaturated with ligands, generating vacant sites and enhancing the diffusion rate of sulfur ions [46].

To further investigate proliferation, the Randles–Sevcik equation was used to quantitatively calculate the diffusion coefficients of different electrodes. The data in Table 1 indicate that the NiMoS catalyst enhances transport capacity of ions, and the ions in the solution exhibit a greater diffusion capacity when utilizing the NiMoS-CF.

The standard rate constants can be used to determine the redox reaction’s kinetics. Higher $k^{0}$ values indicate faster kinetics, reduced energy requirements, and lower overpotential, which are critical for the high-performance RFBs [47]. Reversible electrochemical reactions exhibit high charge transfer rates ($k^{0}$ > 10^−1^ cm s^−1^). Although Nicholson approach is the preferred method for calculating standard rate constants in reversible systems, the Lavagnini modification of Nicholson method in Equation (5) is more suitable for peak-to-peak separation over 200 mV in this experiment. Table 1 presents the results derived from the CV test. The higher redox kinetic parameters suggest that chemical reactions between the redox pairs are more likely to occur, allowing the system to reach equilibrium in a shorter amount of time with NiMoS-CF electrodes [47].

#### 2.2.2. Flow Battery Tests

**Figure 6:**
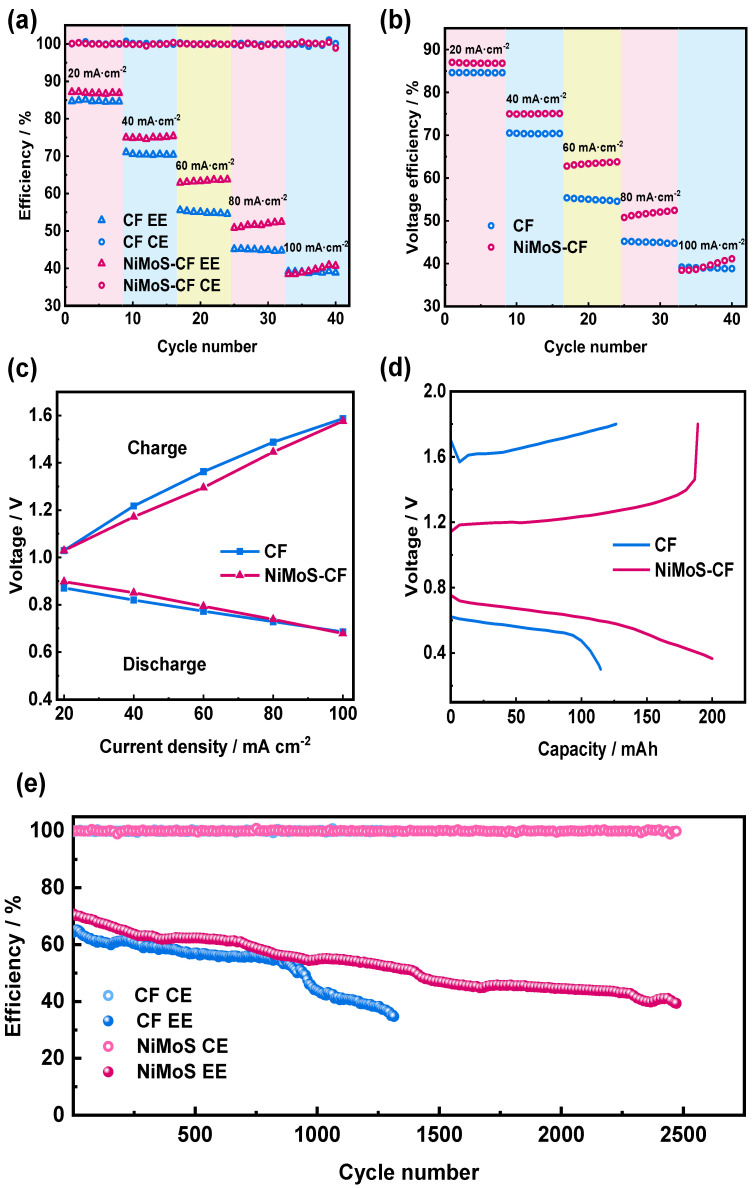
(**a**,**b**) The rate performances of PFRFBs with pristine CF and NiMoS−CF as anode, respectively; (**c**) polarization curves of PFRFBs with pristine CF and NiMoS−CF; (**d**) charge/discharge voltage curves of PFRFBs with pristine CF and NiMoS−CF; (**e**) cell performance of PFRFBs with pristine CF and NiMoS−CF.

To assess the impact of electrodes on battery performance, PFRFBs were constructed with pristine CF and NiMoS-CF electrode, respectively. According to Figure 6a, NiMoS-CF cell exhibited significantly greater energy efficiency than pristine CF cell in the current density ranging from 20 to 100 mA cm^−2^. At higher current densities, NiMoS-CF has a more pronounced positive effect compared to pristine CF serves as an anode. Specifically, the polarization of NiMoS-CF is lower than that of pristine CF RFBs. The energy efficiency is approximately equivalent to that of pristine CF cells when the current density above 100 mA cm^−2^, and a similar trend is observed in the cells’ voltage efficiency (Figure 6b). Consequently, the voltage loss in NiMoS-CF-based PFRFB is reduced as the polarization effect diminishes.

This assumption was supported by the polarization curve test conducted at 50% SOC (Figure 6c). The cell equipped with NiMoS-CF demonstrated lower charge voltage and higher discharge voltage in polarization tests, and the reduction of overpotential will lead to the improvement of EE.

The charging and discharging voltage curves in Figure 6d determine that the charging curve of the pristine CF electrode differs from that of the NiMoS-CF electrode, where the voltage initially decreases and then rises. It is believed that this behavior results from the electrochemical process that occurs when redox ions of various valence states diffuse to the electrode surface. A concentration gradient is created when the concentration difference of ions in the spatial domain decreases because the diffusion rate of ions on the electrode surface is less than the reaction rate. Concentration polarization and related losses are caused by this gradient [48]. At a current density of 100 mA cm^−2^, battery based on NiMoS-CF exhibit lower charge and higher discharge plateaus compared to that based on pristine CF, verifying the battery with NiMoS-CF as the anode has reduced polarization. The curves confirm that NiMoS-CF electrode perform significantly better than pristine CF electrode; Specifically, the electrocatalytic activity of the pristine CF electrode is markedly improved following modification with NiMoS catalyst.

To further assess and compared the characteristics of NiMoS-CF and pristine CF as negative electrodes, the CE and EE of the cells were measured at 40 mA cm^−2^ in Figure 6e. The data revealed that the battery equipped with NiMoS-CF electrode had an energy efficiency (EE) of 71% during the first cycle and 40% after 2500 cycles. That was higher than that of the battery with pristine CF electrode, which had an EE of 64% at the beginning of the cycle and 40% when it became inoperative due to severe capacity degradation after 1300 cycles. It is evident that the battery is capable of maintaining a stable voltage plateau and capacity output over numerous cycles of usage and is not prone to rapid performance degradation.

It is worth noting that the NiMoS-CF electrode’s EE declined after 2500 cycles, owing primarily to CF degradation and electrolyte contamination. During the extended cycle, the CF may corrode or wear mechanically. As the cycle advances, the accumulation of chemical byproducts or impurities in the electrolyte can impede mass transfer, increase overpotential, and result in decreasing efficiency.

When compared to other representative batteries, the PFRFB with NiMoS-CF reported in this paper demonstrates exceptional performance, the capacity retention rate is as high as 99.9%, as illustrated in Table S3. In conclusion, following the modification of the NiMoS catalyst on the pristine CF electrode and subsequent battery testing, the cycle life, EE, and VE values of the battery are significantly enhanced.

## 3. Experimental

### 3.1. Materials

Nickel chloride hexahydrate (NiCl_2_·6H_2_O, AR > 98.0%) was obtained from Shanghai McLean Biochemical Science and Technology Company (Shanghai, China). Sodium molybdate dihydrate (Na_2_MoO_4_·2H_2_O, AR > 98.0%), thiourea (CH_4_N_2_S, AR > 99.0%), ethanol absolute (C_2_H_6_O, AR > 99.5%), potassium ferricyanide (K_3_[Fe(CN)_6_], AR > 99.5%), potassium polysulfide (K_2_S, AR ≥ 40%), and potassium chloride (KCl, AR > 99.0%) were ordered from Sinopharm Chemical Reagent Company (Shanghai, China). The above chemicals were used directly for experiments without any purification. Nafion 212 membrane was provided by Jiangsu Kerun Membrane Materials Company (Suzhou, China). Prior to use, the commercial Nafion 212 was subjected to boiling in a 1.0 M KOH solution at 80 °C for 1 h. This process facilitated the transformation of the membrane from a proton (H^+^) to a potassium (K^+^) form of cation-exchange membrane. Before using, the treated Nafion 212 was soaked in deionized (DI) water.

### 3.2. Preparation of NiMoS Catalyst and NiMoS-CF

The one-step hydrothermal method was used to prepared the NiMoS catalyst and NiMoS-CF electrode. 0.3 mmol L^−1^ NiCl_2_·6H_2_O, 0.3 mmol L^−1^ Na_2_MoO_4_·2H_2_O, and 0.25 mmol L^−1^ of CH_4_N_2_S were added to 100 mL of deionized water. The solution was magnetically agitated at room temperature (~25 °C) until dissolved. Different sizes of carbon felts (1.5 × 1.5 cm^2^ and 2.0 × 2.0 cm^2^) with the above solution were transferred to a 100-mL Teflon-lined autoclave. The reactor was kept in an oven at 180 °C for 24 h. Finally, the NiMoS catalyst and NiMoS-CF were collected, rinsed with DI water, and dried at 60 °C for 10 h before using.

### 3.3. Material Characterizations

The morphologies of pristine CF and NiMoS-CF electrodes were investigated by scanning electron microscopy (SEM, Zeiss GeminiSEM 300, Zeiss, Oberkochen, Germany). The structural and chemical information of the NiMoS-CF electrode were elucidated using high-resolution transmission electron microscopy (HR-TEM, model JEOL F200, JEOL, Tokyo, Japan) at an operating voltage of 200 kV. This analysis included high-angle annular dark-field scanning transmission electron microscopy (HAADF-STEM, JEOL) for imaging, companied by energy-dispersive X-ray spectroscopy (EDS) for elemental mapping. The characteristic diffraction peaks of pristine CF and NiMoS-CF were employed by X-ray diffraction (XRD, D8 ADVANCE and DAVINCI DESIGN, Karlsruhe, Germany) in the range of 3–90° with Cu Kα (λ = 1.5418 Å) radiation, an accelerating voltage of 40 kV and a current of 40 mA. The X-ray photoelectron spectrometer (XPS, ESCALAB250Xi, Thermo Fisher Scientific, Waltham, MA, USA) was performed for high-resolution XPS measurements equipped with Al Kα source. Hydrophilicity of pristine CF and NiMoS-CF were measured by contact angle test (CA, Dataphysics-OCA20, Stuttgart, Germany).

### 3.4. Electrochemical Measurements

Cyclic voltammetry (CV) experiments were conducted within a three-electrode configuration using an electrolyte solution composed of 50 mmol L^−1^ K_2_S and 1.0 mol L^−1^ KCl. The setup included either pristine CF or NiMoS-CF (with dimensions of 1.5 × 1.5 cm^2^) as the working electrode, pristine CF (2 × 2 cm^2^) as the counter electrode, a Ag/AgCl electrode as the reference electrode, with test voltages in the range of −1.3 to 0.3 V and scanning rates between 5 and 50 mV s^−1^ on Chenhua Chi660e Electrochemical Workstation (CH Instrument, Shanghai, China). The electrochemical impedance spectroscopy (EIS) measurements were done in the amplitude of 15 mV and the frequency range of 10 MHz to 100 kHz. The glassy carbon electrode (GCE) was used in the same electrolyte system as working electrode, which was drop-coated with a catalyst ink of 3 mg NiMoS + 2 mg carbon powder + 80 µL Nafion + 900 µL anhydrous ethanol. S/Fe RFB cells (Figure S1) were fabricated using pristine CF or NiMoS-CF as the anode (2 × 2 cm^2^) and pristine CF (2 × 2 cm^2^) as the cathode, separated by a Nafion 212 ion-exchange membrane. The anolyte was prepared as a 40 mL aqueous solution containing 2.0 mol L^−1^ K_2_S and 2.0 mol L^−1^ KCl, while the catholyte consisted of 40 mL of 1.0 mol L^−1^ K_3_[Fe(CN)_6_] and 2.0 mol L^−1^ KCl. Both electrolytes were circulated through the cell at a constant flow rate of 70 mL min^−1^ during electrochemical testing. Tests of rate performance and long-term charge/discharge cycle were conducted on LAND battery test system (CT3002K, LAND Electronics, Wuhan, China).

At current densities ranging from 20 to 100 mA cm^−2^, the rate performance at 50% State of Charge (SOC) was determined. For each specified current density, a series of eight charge/discharge cycles were executed to record the values of Coulombic Efficiency (CE), Voltage Efficiency (VE) and Energy Efficiency (EE). In constant current mode, long-term charge/discharge cycling experiments were conducted at 40 mA cm^−2^ within the voltage in the range of 0.3 to 1.8 V. Polarization curves were obtained on the same device.

### 3.5. Electrochemical Calculation

The diffusion coefficients for different electrodes were quantified according to the Randles-Sevcik equation [49]:

(1) $$I_{p}=0.4463nFAC{\left(\frac{nFvD}{RT}\right)}^{\frac{1}{2}}$$

Or if the solution is at 25 °C [50]:

(2) $$I_{p}=269000n^{\frac{3}{2}}ACD^{\frac{1}{2}}V^{\frac{1}{2}}$$

where $I_{p}$ represents the peak current in amperes, n denotes the number of electrons involved in the redox process (commonly 1), D is the diffusion coefficient (cm^2^ s^−1^), V is the scan rate (V s^−1^), A is the electrode area (cm^2^), and C is the concentration of the substance to be measured (mol cm^−3^).

The Nicholson approach [51] was used to calculate standard rate constants k^0^ for various electrodes.

(3) $$\psi =\frac{{\left(\frac{D_{O}}{D_{R}}\right)}^{\alpha /2}k^{0}}{{\left(\frac{\pi D_{O}nFv}{RT}\right)}^{1/2}}$$

The dimensionless kinetic parameter, denoted as ψ, is derived from ΔEp [52], where R is the transfer coefficient, n is the number of electrons transferred in the redox process, F is the Faraday constant. R and T have their usual meanings. Typically, it is reasonable to assume that the diffusion coefficients for both the oxidized ($D_{O}$) and reduced ($D_{R}$) forms of the mediator are nearly identical, and the reduction and oxidation kinetics are relatively symmetrical (R ∼ 0.5) [53].

From Equation (3), the slope of a plot of ψ versus $\upsilon^{1/2}$ can be used to determine $k^{0}$. The values of ψ at different peak separations can be estimated practically using Equation (4).

(4) $$\psi =\frac{\left(-0.6288+0.0021\Delta Ep\right)}{1-0.017\Delta Ep}$$

where ΔEp is the redox peak current potential difference (V).

The Lavagnini method [54] is an improvement on the Nicholson method and can achieve peak-to-peak separation above 200 mV which is more suitable for this experiment:

(5) $$\psi =2.18{\left(\beta /\pi \right)}^{1/2}exp[-{(\beta }^{2}F/RT)n∆Ep]$$

where β is the transfer coefficient for the electrode process [55].

ECSA is the Electrochemical Active Surface Area, a higher ECSA value implies that the electrode’s effective area is greater.

(6) $$ESCA=\frac{C_{dl}}{C_{s}}A$$

where $C_{dl}$ is the double capacitive electric layer, $C_{s}$ is the differential capacitance of the smooth metal, which is taken at 0.04 mF cm^−2^ in this research [56], and A is the geometric surface area of the electrode.

In this experiment, all currents in non-faradic region were assumed to be non-faradic currents. The region for non-faradic is −0.1–0 V (vs. KCl saturated Ag/AgCl). The current i is obtained at the middle point of potential range from CV curves at 0.01–0.1 V s^−1^. And of the system is taken as the average of absolute value of charging current. The electrolyte used in ECSA tests is 0.05 M K_2_S with 1.0 M KCl.

## 4. Conclusions

In summary, we have successfully fabricated NiMoS catalyst on the carbon felt electrode via a one-step hydrothermal method. The electrochemical reaction kinetics and charge transfer behaviors of the obtained NiMoS-CF electrode were improved by the NiMoS modification, resulting in the accelerated kinetics of polysulfide redox reactions. The integrated PFRFB with NiMoS-modified carbon felt electrode as the anode, exhibited a significant improvement in the energy density and cycle stability, including an energy efficiency of 70%, voltage efficiency of 87%, outperforming the pristine CF-based battery with corresponding values of 64% and 84%, respectively. Furthermore, the cycle life of the PFRFB was extended to 2500 cycles with a coulombic efficiency consistently maintained above 99% at 40 mA cm^−2^. This research provides a strategy for enhancing the performance of the S/Fe RFBs by employing sulfide bimetallic catalyst as the electrode booster, offering a promising direction for developing the cost-effective and high-performance energy storage systems.

Furthermore, while the three-electrode test described in this study can be utilized for high-precision electrochemical measurements, it is susceptible to effects from solution resistance, voltage window, and temperature. To avoid such impacts in future work, the test error will be addressed to the greatest extent possible.

## Figures and Tables

**Table 1:** Diffusion rates of different electrodes.

Electrodes	Pristine CF	NiMoS-CF
D/(cm^2^ s^−1^)	9.42 × 10^−5^	1.597 × 10^−4^
k^0^/(cm s^−1^)	1.3 × 10^−3^	1.2 × 10^−3^
C_dl_/(mF cm^−2^)	6.44	7.02
ECSA/cm^2^	362.25	394.875
Tafel Slope/(mV dec^−1^)	153.1	48.91

## Data Availability

Data will be made available on request.

## Supplementary Materials

The following supporting information can be downloaded at: https://www.mdpi.com/article/10.3390/molecules30061219/s1; Figure S1: Photograph of the NiMoS-S/Fe cell stack; Figure S2: XRD pattern of NiMoS catalyst; Figure S3: (a) XPS survey spectrum of NiMoS catalyst; high-resolution spectra for (b) Ni 2p, (c) Mo 3d and (d) S 2p spectra of NiMoS catalyst; Figure S4: Glassy carbon electrode with NiMoS catalyst; Figure S5: CV curves of the original GCE and NiMoS modified GCE at 10 mV s^−1^; Table S1: Kinetic parameters of S_2_^2−^/S^2−^ from CF and NiMoS-CF’s CV data. Related to Figure 4a,b; Table S2: Fitting results for the as-prepared samples from the Nyquist plots; Table S3: An overall comparison with other representative RFBs. References [10,13,16,27,31,32,42,46,57,58,59,60,61] are cited in the Supplementary Materials.
