# Supplementary material for: NiMoS-Modified Carbon Felt Electrode for Improved Efficiency and Stability in a Neutral S/Fe Redox Flow Battery
Source: Molecules. 2025 Mar 8;30(6):1219. doi: 10.3390/molecules30061219 (PMC11944520; doi:10.3390/molecules30061219)
Supplement: Supplementary file 1 [file molecules-30-01219-s001.zip › molecules-3515616-supplementary.pdf]

Supplementary Material

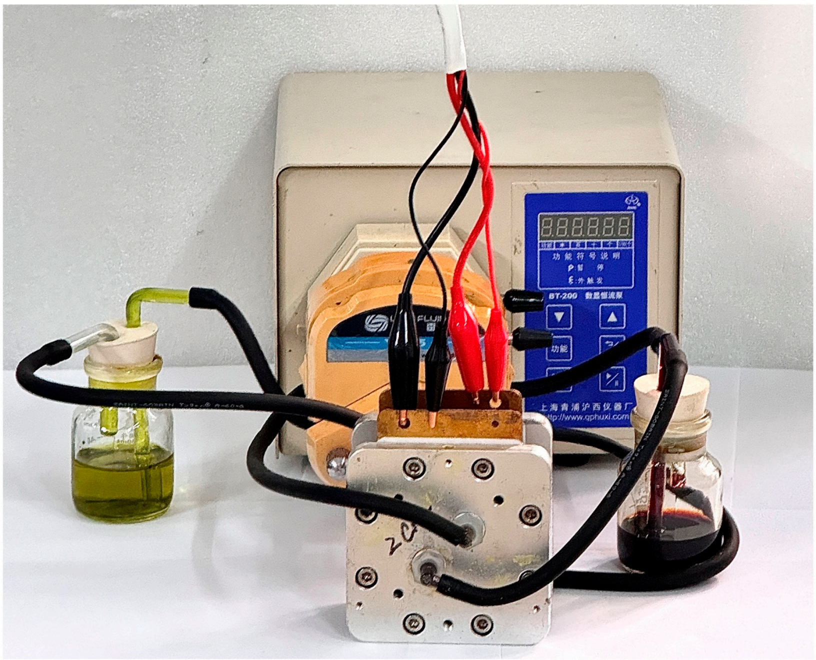

Figure S1. Photograph of the NiMoS-S/Fe cell stack.

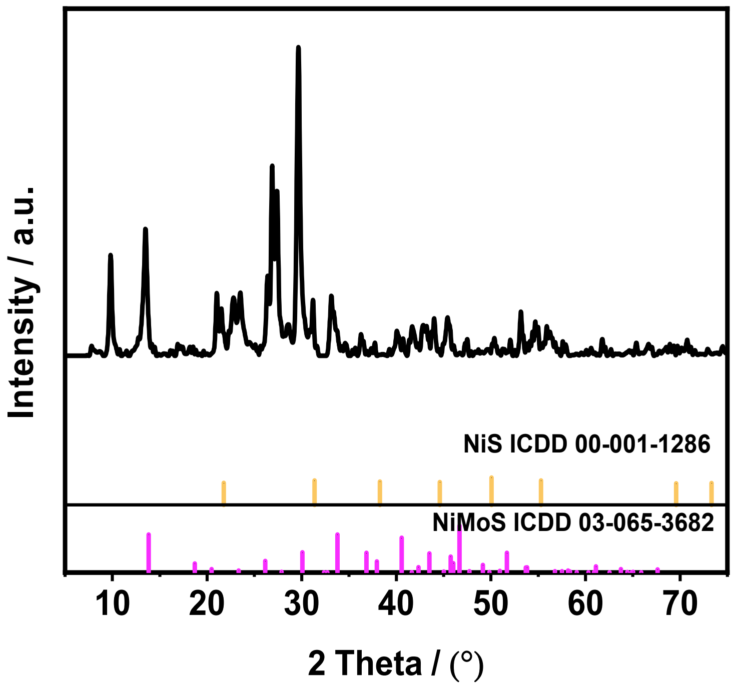

Figure S2. XRD pattern of NiMoS catalyst.

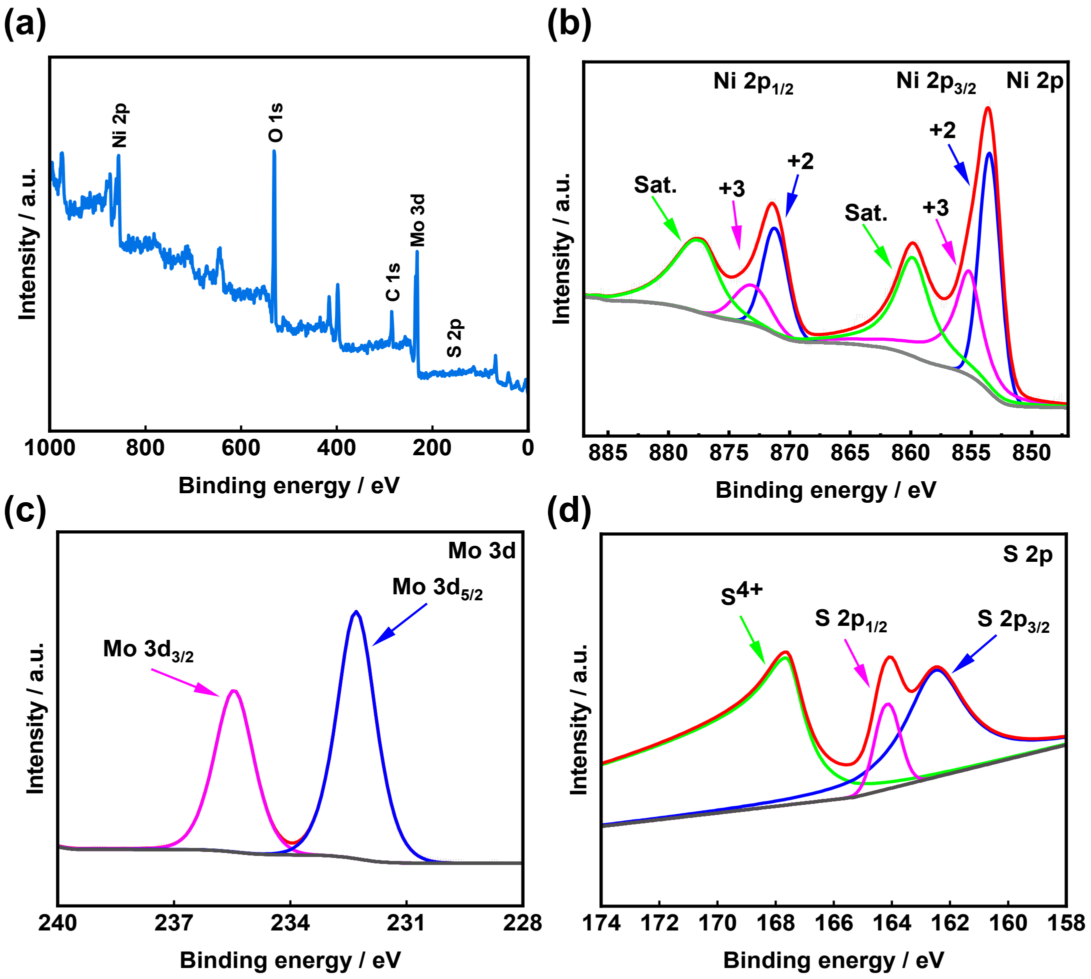

Figure S3. (a) XPS survey spectrum of NiMoS catalyst; High-resolution spectra for (b) Ni 2p, (c) Mo 3d and (d) S 2p spectra of NiMoS catalyst.

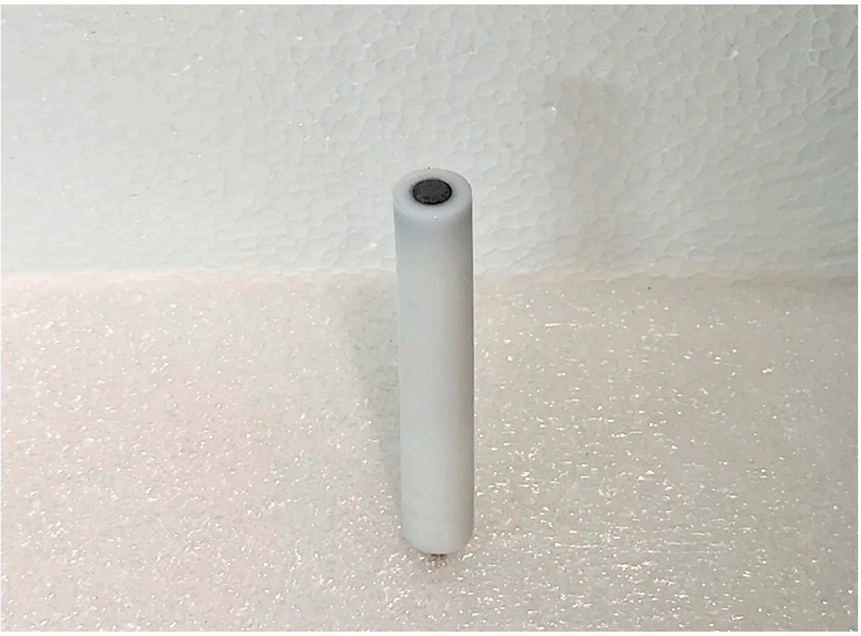

Figure S4. Glassy carbon electrode with NiMoS catalyst.

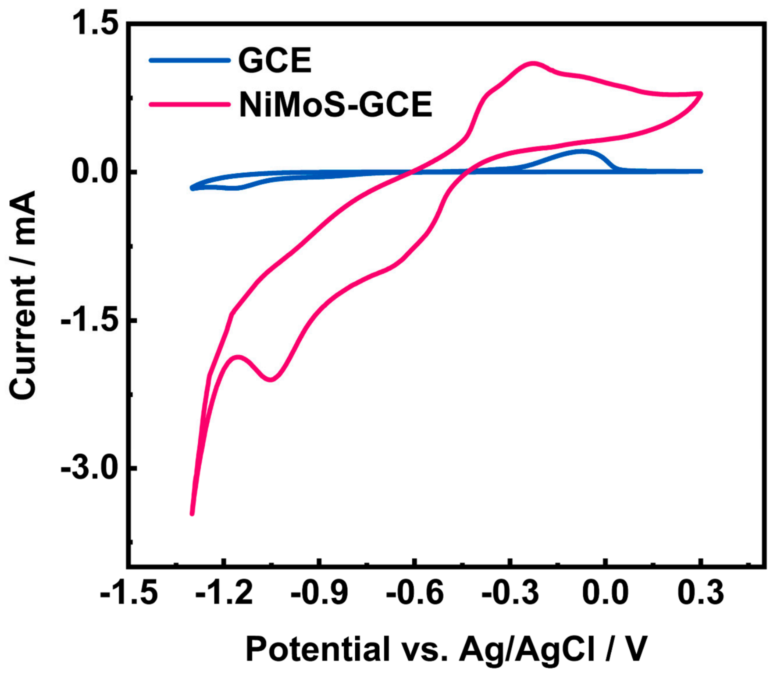

Figure S5. CV curves of the original GCE and NiMoS modified GCE at 10 mV s^{-1}.

Table S1. Kinetic parameters of S_2^{2-}/S^{2-} from CF & NiMoS-CF's CV data. Related to Fig. 4(a) and(b).

v/mV s^{-1}	E_{pa} (V)	E_{pc} (V)	i_{pa} (A)	i_{pc} (A)	$\lvert i_{pa}/i_{pc} \rvert$
5	-0.125	-0.841	0.061	-0.04604	1.32493
10	-0.023	-0.95	0.08308	-0.06684	1.24296
20	0.086	-1.082	0.115	-0.1009	1.13974
30	0.174	-1.187	0.1362	-0.1243	1.09573
40	0.224	-1.254	0.1481	-0.1392	1.06393
50	0.273	-1.298	0.1581	-0.152	1.04013

v/mV s^{-1}	E_{pa} (V)	E_{pc} (V)	i_{pa} (A)	i_{pc} (A)	$\lvert i_{pa}/i_{pc} \rvert$
5	-0.083	-0.809	0.07769	-0.05852	1.32758
10	0.057	-0.887	0.1082	-0.08881	1.218331
20	0.216	-0.995	0.139	-0.1211	1.147812
30	0.291	-1.076	0.1534	-0.1415	1.084099
40	0.299	-1.146	0.1653	-0.1591	1.038969
50	0.3	-1.218	0.1736	-0.1734	1.001153

Table S2. Fitting results for the as-prepared samples from the Nyquist plots.

Electrodes	Rs (mΩ)	Rct (mΩ)
CF	3169	3768
NiMoS-CF	3092	524

Table S3. An overall comparison with other representative RFBs.

Electrode	RFB Types	Electrolyte	Membrane	Current density [mA cm^{-2}]	Energy efficiency [%]	Energy density [Wh L^{-1}]	Capacity retention [%]	Ref.
NiMoS catalyst-modified carbon felt	Polysulfide/ferricyanide flow batteries (S/Fe RFBs)	2.0 M K2S + 1.0 M KCl	Nafion 212	40	70.34	30.1514	99.9425	This Work
Carbon felt with a piece of iron foil	All-iron hybrid redox flow batteries	1.0 M FeCl2·4H2O + 1.0 M C2H5NO2 + 1.0 M NaBr + DES	Nafion-Na+	10	54	-	-	[10]
OHP-Co/NC-850	polysulfide/ferrocyanide RFB	2.0 M Na 2S2	Nafion 115	40	69.84	-	99.7	[13]
Graphite electrodes	Polysulfide-ferricyanide flow battery (PSFRFB)	4.0 M K2Sx + 2.0 M KCl	PVDF-co-HFP based CEM	40	63	-	88	[16]
Co-graphite felt	Ferri/ferrocyanide - polysulfide (Fe/S) flow battery	0.05 M Na2S2 + 1.0 M NaCl	Nafion 117	20	74	-	98	[27]
CuS catalyst-modified carbon felt	Polysulfide-ferricyanide flow battery (S/Fe RFBs)	2.0 M K2S + 1.0 M KCl	DS57-K	50	75.41	-	99.54	[31]
Carbon felt	Polysulfide/ferricyanide redox flow battery (S/Fe RFBs)	2.0 M K2S + 1.0 M KCl	Nafion 212	20	80.97		96.9	[32]
WS2 catalyst-modified carbon felt	Polysulfide/ferricyanide flow batteries (S/Fe RFBs)	2.0 M K2S + 1.0 M KCl	Nafion 212	40	84	-	96	[42]
Carbon felt	Neutral aqueous flow	1.0 M K2S + 1.0 M KCl	Nafion 212	30	-	92.8	181.8	[46]

	battery							
TCP and copper-plated GF	Vanadium-Iron redox flow battery	0.1 M Fe^{2+} + 0.1 M V^{3+} + 45 ml DES	Nafion 212	2	91.8	9.6	-	[57]
PAN-based Sigracell battery felt	Non-aqueous redox flow battery (NAqRFB)	Acetonitrile + 0.1 M $TEABF_4$ + 0.05 M Fc	Nafion 117	14	56.91	13.5	-	[58]
-	Non-aqueous vanadium redox flow battery	0.01 M $V(acac)_3$ + 0.1 M TEABF 4/ACN	L-PSQ/Celgard composite membrane	1.39	43.7	-	-	[59]
Zinc sheet	Non-aqueous redox flow batteries	0.05 M $(NH_4)_2[Ce(NO_3)_6]$ + 0.5 M Zn $(OTf)_2$/ACN	Nafion XL	2	64	24.3	-	[60]
Ni foam	Alkaline S/Fe RFB	6.0 M K_2S + 0.5 M KOH	Nafion 212	100	60	48.88	93.15	[61]
